# Superior Multiplexing Capacity of PlexPrimers Enables Sensitive and Specific Detection of SNPs and Clustered Mutations in qPCR

**DOI:** 10.1371/journal.pone.0170087

**Published:** 2017-01-23

**Authors:** Lit Yeen Tan, Samantha Michelle Walker, Tina Lonergan, Nicole Elizabeth Lima, Alison Velyian Todd, Elisa Mokany

**Affiliations:** SpeeDx Pty Ltd, Eveleigh, NSW, Australia; Institute of Genetics and Developmental Biology Chinese Academy of Sciences, CHINA

## Abstract

**Background:**

Whilst qPCR provides an extremely powerful tool for genetic analysis, some applications such as multiplexing variant alleles (eg SNPs, point mutations or deletions), remain challenging using current primer/probe systems. The novel design features of PlexPrimers allow sensitive, multiplexed analysis of variant alleles even when these are tightly clustered.

**Method:**

PlexPrimers were combined with PlexZymes in qPCR assays for the detection of SNPs in human absorption, distribution, metabolism, and excretion (ADME) genes; clustered mutations in the 23S rRNA gene which confer antibiotic resistance to *Mycoplasma genitalium*; and deletions within the human epidermal growth factor receptor (EGFR) gene.

**Results:**

The combination of PlexPrimers and PlexZymes allowed robust multiplexing of targets which resulted in 100% concordance with results obtained using hydrolysis probe kits for 14 SNPs in the ADME genes. A 7-plex qPCR assay targeting *M*. *genitalium*, 5 clustered mutations associated with macrolide resistance and an internal control, allowed efficient amplification of all targets, with all 5 mutations detected in a single channel. Finally, the strategy was employed to analyse common EGFR mutants with high sensitivity, detecting deletions present at only 0.01%.

**Conclusion:**

PlexPrime is a novel technology for the detection of genetic variants. Unlike previous strategies, the combination of PlexPrimers with PlexZymes enables both allele-specific detection and allele-specific amplification in qPCR. The study demonstrated highly sensitive and specific detection of mutations and SNPs, and superior multiplexing capacity. The ability to multiplex clustered genetic variants reduces the time to result providing more actionable information.

## Introduction

Real-time quantitative PCR (qPCR) is a powerful diagnostic technique which is widely used to detect and characterize sequences associated with human, animal or plant diseases. Assays may be designed to detect targets, such as viral or bacterial sequences to identify the cause of an infection, or alternatively they may be designed to distinguish specific sequence variations within genomes. These variations include inherited single nucleotide polymorphisms (SNPs) or acquired somatic mutations such as point mutations, deletions and insertions which are frequently associated with cancer. In bacteria, mutations may be associated with antibiotic resistance. Detection and characterisation of genomes affords broad clinical utility. It can assist in diagnosing disease, predicting response or resistance to therapy, determining prognosis, and allowing longitudinal disease monitoring. Multiplex qPCR is highly advantageous since it enables the analysis of multiple targets simultaneously, thereby increasing the amount of information per specimen and further allowing internal controls to be built in. Specific examples of targets of diagnostic significance include SNPs within ADME genes, which can affect drug efficacy and toxicity; mutations in the 23S rRNA gene of *Mycoplasma genitalium* which can confer resistance to azithromycin often used to treat sexually transmitted infections (STIs); and mutations in the EGFR gene which can predict response to cancer therapy.

PCR methods for allelic discrimination of SNPs and mutations employ various strategies. These include amplification by allele-specific primers, detection by allele-specific probes and/or determination of melting temperature profiles [1–3]. The Amplification Refractory Mutation System (ARMS), Mismatched Amplification Mutation Assay (MAMA), SuperSelective and Dual Priming Oligonucleotide (DPO) methods all employ “allele-specific primers” which target the amplification of specific variants that are complementary to their 3’ termini [4–7]. Resultant amplicons may be detected in real-time using generic hydrolysis probes or Molecular Beacons, which hybridize to a region which does not include the variant base [8,9]. Scorpion probes which are primer-probe hybrids can also use an ARMS approach for allele-specific amplification and detection [10,11]. However, since SNPs are variations in the same position, and mutations are often tightly clustered in hotspots of functional and structural importance, primer competition and cross-priming makes it difficult to develop multiplex assays using these approaches. Alternatively sequence variants can be amplified by generic primers, which do not selectively bind to the specific variant, and amplicons can be subsequently detected and distinguished using “allele-specific probes” [12,13]. However, similar to the limitation with allele-specific primers, competition between allele-specific probes limits the number of clustered variants that can be analysed in a single reaction. Further, competition between either allele-specific primers or allele-specific probes generally decreases both specificity and sensitivity of detection. High resolution melt (HRM) analysis avoids this by differentiating on the basis of melt curve characteristics of specific alleles [12], but not all variants can be detected with equal ease and sensitivity. The method has greater difficulty distinguishing particular changes, for examples A to T and C to G, and vice versa, which result in relatively minor differences in the melting temperatures. Overall it remains challenging to sensitively and specifically detect variant alleles in a multiplex context using any of the above approaches.

**Fig 1:**
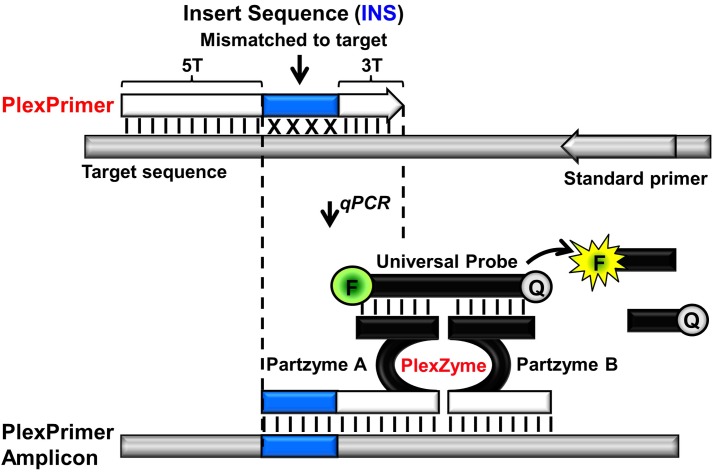
PlexPrimer amplification coupled with PlexZyme detection in qPCR. The PlexPrimer contains three functional regions; a 5’ target recognition region (5T); a short 3’ target-specific sequence (3T) and an intervening Insert Sequence (INS) region which is mismatched with respect to the target. PlexPrimers can be designed to be allele-specific by matching the termini of the 3T region to the target variant. During amplification, the INS is incorporated into the PlexPrime amplicons and this can be detected in real-time using PlexZymes. PlexZymes are nucleic acid enzymes which form from their component partzymes, A and B, only when target amplicons are present. Each of the partzymes contain a probe binding arm, a partial catalytic core and a target binding arm, orientated such that partzyme A binds to the amplicon in the region that includes the complement of the INS and the 3T region (between dotted lines), and Partzyme B binds adjacently downstream. Catalytically active PlexZymes bind and cleave universal reporter probes between fluorophore (F) and quencher (Q) moieties resulting in signal generation.

This paper describes a new method for discrimination of variant sequences, which circumvents the limitations discussed above. The strategy combines allele-specific primer amplification using PlexPrimers with allele-specific detection using PlexZymes (also known as MNAzymes) [14,15]. The novel feature of PlexPrimers is that each contains an “insert sequence” (INS), which is non-complementary to the target initially but which is introduced into amplicons during amplification (Fig 1). The INS is positioned between 5’ and 3’ target-specific regions denoted as 5T and 3T respectively. For multiplexed mutation detection, each PlexPrimer contains a different INS and is designed to be allele-specific via complementarity of the 3’ terminus of the 3T region with the target mutation. This strategy provides multiple advantages. Firstly, when the PlexPrimer binds initially, the INS effectively results in a shortening of the sequence at the 3’ end of each primer which is matched to the target. This increases the pressure for the polymerase to only extend primers matched at their termini, and in turn, this promotes highly stringent, selective amplification of specific mutant alleles. Secondly, the presence of unique INSs in the amplicons increases the ability to simultaneously multiplex targets since they reduce competition between allele-specific PlexPrimers. Finally, the INSs enhance the specificity of detection by reducing competition between allele-specific PlexZymes in real-time. This is achieved by designing each PlexZyme to have amplicon sensing regions that bind to the mutation, the INS and downstream target sequence. This paper demonstrates features and applications of this novel method and provides examples of their use in several settings where they provide advantages.

## Materials and Methods

### Standard qPCR conditions

Unless specified otherwise, the qPCR mixes contained 8 mM MgCl_2_, 200 μM of each dNTP, 1x Immobuffer (Bioline) and 2 U MyTaqHS™ DNA polymerase (Bioline) in 25 μL final volume in 96 well plates on the CFX96™ (Bio-Rad Laboratories). These reactions were thermocycled at 95°C for 2 min followed by 10 cycles of 95°C for 15 s, 61°C for 60 s (-1°C per cycle) and 50 cycles at 95°C for 15 s, 52°C for 60 s.

### ADME SNP genotyping

#### Templates

Fourteen ADME SNPs were genotyped using human genomic DNA (gDNA) templates listed in Table 1.

**Table 1:** DNA templates & comparator assays for analysis of ADME SNPs.

Gene dbSNP ID	Allele #/base	DNA template used for testing SNPs		Comparator TaqMan SNP Genotyping Assay^~^
		Homozygous	Heterozygous	
TPMT rs56161402	1/C	NA11830*	nt	C_____19569_20
	2/T	nt		
CYP2C9 rs1799853	1/C	NA18992*	NA12248*; IM-9^#^	C__25625805_10
	2/T	nt		
CYP2C19 rs4244285	1/A	NA12891*; Calu-1^#^	NA18855*; NA19003*	C__25986767_70
	2/G	NA11830*; NA18970*		
CYP2C19 rs4986893	1/A	nt	NA18573*; NA18948*	C__27861809_10
	2/G	NA18970*; NA18992*		
CYP2D6 rs5030655	1/del	nt	nt	C__32407243_20
	2/T	NA11830*		
CYP2D6 rs1065852	1/C	NA12248*; NA18608*; NA18855*; NA18992*; NA19003*	NA06993*; NA11830*; NA18573*; NA18871*; NA18970*	C__11484460_40
	2/T	NA18948*; IM-9^#^		
SLC22A2 rs316019	1/G	NA18608*; NA18855*	IM-9^#^	C___3111809_20
	2/T	nt		
SLCO1B3 rs4149117	1/G	NA18573*; NA18948*	NA06993*; NA18608*	C__25639181_40
	2/T	NA18855*; NA18992*		
CYP2C8 rs11572103	1/A	nt	nt	C__30634034_10
	2/T	NA11830*		
CYP2C8 rs1058930	1/C	NA18871*; NA18948*	NA12248*; NA12891*	C__25761568_20
	2/G	Calu-1^#^		
CYP2A6 rs1801272	1/A	NA11830*	nt	C__27861808_60
	2/T	nt		
DPYD rs1801265	1/C	NA18855*; NA18871*	HT-29^#^; SW480^#^	C___9491497_10
	2/T	NA18608*; Calu-1^#^		
CYP1B1 r s1056836	1/C	NA18871*; SW480^#^	NA12248*	C___3099976_30
	2/G	NA12891*; NA18948*		
ABCB1 rs2032582	1/T	nt	T/C NA19003; C/A NA06993; T/A NA18970	C_11711720D_40 C_11711720C_30
	2/C	NA18855; NA18871		
	3/A	NA18573; NA18608		

nt—not tested.

* Human genomic DNA (gDNA) supplied by Coriell Cell Repositories.

^#^ Human gDNA extracted from ATCC cell lines using the QIAamp DNA and Blood Mini Kit (Qiagen) using manufacturer’s instructions.

~ TaqMan^®^ Drug metabolism genotyping assays (Thermo Fisher Scientific).

#### Oligonucleotides

The sequence of primers, partzymes and probes for the ADME assays are listed in Table A in S1 File for the bi-allelic assays and Table 2 for the tri-allelic assay. Bi-allelic multiplexed reactions contained oligonucleotide SNP components that were required for allele 1 and allele 2, or for both. Tri-allelic reactions contained additional SNP oligonucleotides that were required for the third allele. The oligonucleotide concentrations in a multiplex reaction were 400 nM of the Reverse primer and 400 nM of Partzyme B and for each allele in the reaction there was 40 nM Forward primer, 100 nM Partzyme A, and 200 nM Probe.

**Table 2:** Oligonucleotides for analysis of a tri-allelic ADME SNP (dsSNP ID rs2032582) in a single multiplex reaction.

**SNP**	**Type of oligo**	**Sequence (5'to 3')^**	Modifications*
Allele 1	Forward primer	*TAATCAATCATATTTAGTTTGACTCA***AGACATACTA***CTTCCC***T***G****A***	-
	Partzyme A	**AGACATACTA***CTTCCC***T***G**A**A*ACAACGAGAGGCGTGAT	3' phosphate
	Probe 5	ATCACGCCTCguCCCCAGCTC	5' F; 3' FQ
Allele 2	Forward primer	*TCTACTTAATTAATCAATCATATTTAG***CACAATGATG***CTTCCC***T***G****T***	-
	Partzyme A	**CACAATGATG***CTTCCC***T***G****T****A*ACAACGAGAGGAAACCTT	3' phosphate
	Probe 2	AAGGTTTCCTCguCCCCAGCTC	5’ T; 3’ RQ
Allele 3	Forward primer	*AATACTTTACTCTACTTAATTAATCAA***GATTCGAGAA***CTTCCC***C***G****C***	-
	Partzyme A	**ATTCGAGAA***CTTCCC****C****G****C****A*ACAACGAGAGGGAGGAG	3' phosphate
	Probe 6	CTCCTCCCTCguCCCCAGCTC	5’ Q; 3’ B2
Alleles 1, 2, 3	Reverse primer	*ATGTTGTCTGGACAAGCACTGAAA*	-
	Partzyme B	GAGCTGGGGAGGCTAGC*TCCTTCTAGTTCTTTCTTATCTTTC*	3' phosphate

^ Ribonucleotide bases are in lower case and deoxyribonucleotide bases are in uppercase. The sequence complementary to the target is in italics, the variant alleles are in bold and italics, the mismatch is bold and the INS are bold and underlined. The other sequence comprises the rest of the PlexZyme, with underline denoting the probe binding sequence.

* Fluorophores used to label Probes are 6-FAM (F), Texas Red (T), and Quasar 705 (Q). Quenchers attached to the Probes are Black Hole Quencher 2 (B2), Iowa Black^®^ FQ (FQ) and Iowa Black^®^ RQ (RQ).

#### PlexPrime/PlexZyme qPCR assay

Standard qPCR conditions were used with the addition of 10 U of Ribosafe (Bioline) to the qPCR mix. Each reaction contained 5 ng of gDNA template or no-DNA.

#### TaqMan® drug metabolism genotyping assays

TaqMan^®^ Drug metabolism genotyping assays (Thermo Fisher Scientific) (listed in Table 1) are commercial hydrolysis probe kits that were also used to genotype ADME SNPs. These were performed according to manufacturer’s instructions. Template DNA (5 ng) or no-DNA was added to each reaction. The reactions were performed in 25 **μ**L final volume in 96 well plates on the CFX96™ (Bio-Rad Laboratories) and thermocycled at 95°C for 10 min followed by 50 cycles of 95°C for 15 s and 60°C for 60 s.

### *M*. *genitalium* and mutations associated with azithromycin resistance

#### Templates

The MG 23S Positive Control kit (beta version) (SpeeDx Pty Ltd; Australia) contained quantitated synthetic DNA for the analysis of the *M*. *genitalium* MgPa and 23S rRNA genes (wild type A2058 and A2059 or mutant A2058G, A2058C, A2058T, A2059G, or A2059C *Escherichia coli* numbering) and an internal control template.

#### PlexPrime/PlexZyme qPCR assay

The MG 23S assay (beta version) (SpeeDx Pty Ltd; Australia) contained all the components for the multiplexed amplification and detection of *M*. *genitalium* (MgPa gene) and five mutations in the 23S rRNA gene (A2058G, A2058C, A2058T, A2059G and A2059C) and the internal control. The assay was used according to the manufacturer’s instructions in 20 **μ**L final volume in 96 well plates run on the LC480 II (Roche Diagnostics). Reactions were thermocycled at 95°C for 2 min, followed by 10 cycles of 95°C for 5 s, 61°C for 30 s (-0.5°C per cycle), and 40 cycles of 95°C for 5 s, 52°C for 40 s. Ten-fold serial dilutions of 10^6^ to 10^2^ copies of MgPa and 23S rRNA templates in a background of 10^4^ copies human genomic DNA (gDNA) (Promega) and internal control template were analysed. Control reactions, containing 10^6^ copies of wild type (A2058 and A2059) template in a background of 10^4^ copies human genomic DNA or no-DNA, were also performed. Analysis was performed according to the manufacturer’s instructions.

### EGFR deletions

#### Template

Plasmid templates were used to analyse EGFR deletions within exon 19, specifically c.2235-2249del and c.2236-2250del. Human wild type gDNA was extracted from the ATCC cell line A549 using the QIAamp DNA and Blood Mini Kit (Qiagen) according to the manufacturer’s instructions.

#### Oligonucleotides

The sequences of primers, partzymes and the probe for amplification and detection of EGFR deletions c.2235-2249del and c.2236-2250del are listed in Table 3 along with the concentration used.

**Table 3:** PCR primers, partzymes and probes for EGFR deletion assay.

Type	Sequence (5'to 3')^	Modifications*	Concentration
Probe 1	ACCGCACCTCguCCCCAGCTC	5’ T, 3’ RQ	200 nM
Forward primer	*GAGAAAGTTAAAATTCCCGT***TCAATACCAT***GCTATCAAAAC*		40 nM
Reverse primer	*CAGACATGAGAAAAGGTGGGC*		200 nM
Partzyme A	**AATACCAT***GCTATCAAAACATCTCA*CAACGAGAGGTGCGGT	3' phosphate	100 nM
Partzyme B	GAGCTGGGGAGGCTAGCT*CGAAAGCCAACAAGGAAATCC*	3' phosphate	200 nM

^ Ribonucleotide bases are in lower case and deoxyribonucleotide bases are in uppercase. The sequence complementary to the target is in italics, the INS are bold and underlined and the other sequence comprises the rest of the PlexZyme, with underline denoting the probe binding sequence.

* Fluorophore used to label the Probe is Texas Red (T) and the quencher attached to the Probe is Iowa Black^®^ RQ (RQ).

#### PlexPrime/PlexZyme qPCR assay

Standard qPCR conditions were used to analyse the c.2235-2249del and c.2236-2250del DNA plasmids (10^4^ 10^3^, 10^2^ and 10 copies) diluted in a background of 10^4^ copies of wild type A549 human gDNA thus producing reactions containing 100%, 10%, 1% and 0.1% of each deletion mutants respectively. In parallel 10^4^ copies of wild type A549 gDNA was analysed to determine specificity. To further test the detection limit of the plasmids (10^3^, 10^2^ or 10 copies) were diluted in 10^5^ copies of A549 gDNA producing reactions containing 1%, 0.1% and 0.01% mutant alleles, respectively. Wild type gDNA (10^5^ copies) and no-DNA controls were analysed in parallel.

## Results

### Improved strategy for multiplex analysis of SNPs and mutations

**Fig 2:**
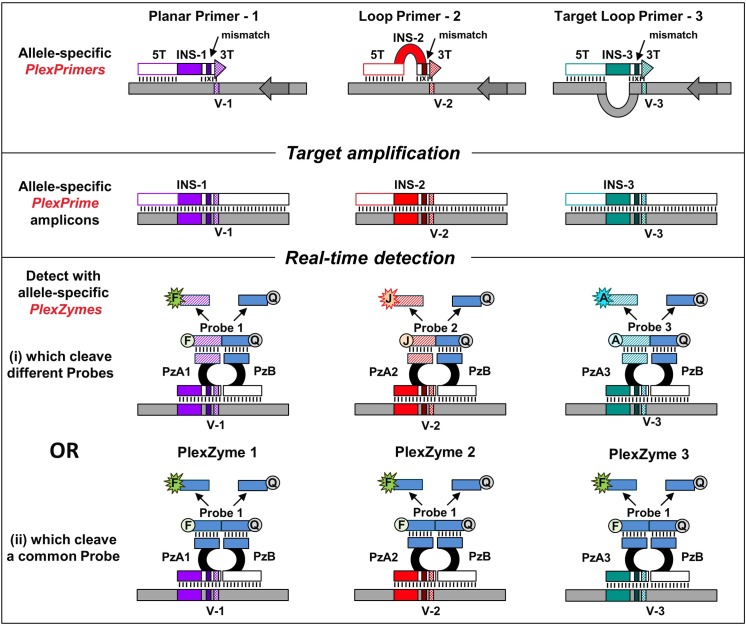
Allele-specific amplification coupled with allele-specific detection. In multiplex qPCR, each allele-specific PlexPrimer has three regions, namely a 3T region that matches the variant base (V-1, V-2 or V-3) and has a single introduced mismatch; a unique INS (INS-1, INS-2 or INS-3) which is mismatched with the target and which can adopt either a Planar, a Loop or a Target Loop conformation; and a 5T region that can bind to different upstream sequences as a result of alternative conformations of the INS. Each variant amplicon is detected by an allele-specific PlexZyme. For example, PlexZyme 1 which targets the V-1 amplicon, is composed of partzyme A1 (PzA1) and partzyme B (PzB). The target arm of PzA1 specifically targets the V-1 base and INS-1 in the amplicon. For allele-specific detection and identification (i), allele-specific amplicons are detected using different hybrid probes, wherein half of each probe is the same and binds to the common PzB and the remaining unique half binds to either PzA1, PzA2 or PzA3. Each hybrid probe is labelled with a different fluorophore denoted F, J or A, respectively, allowing distinct readouts. Alternatively allele-specific amplicons may be detected by PlexZymes using a common universal probe (ii), where the probe binding arm of PzA is the same for each PlexZyme matched to the variants.

**Fig 3:**
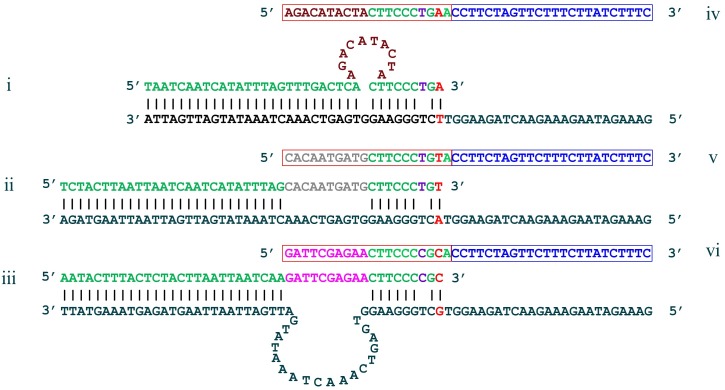
Oligonucleotide components used in the single well triplex assay for ABCB1 rs2032582. Multiplexed PlexPrimers are designed in i) Loop, ii) Planar and iii) Target Loop confirmations to reduce competition and produce amplicons that can then be easily distinguished by PlexZymes due to their substantial sequence difference. Only the partzyme target sensor arms of each PlexZyme are illustrated here, showing partzyme A sensor arms (red boxes) which are specific for each variant allele T, A and G (iv, v, vi respectively), and partzyme B sensor arms (blue boxes) which are common to all variant alleles. The additional single mismatch in the primer 3T region is in purple. The target sequence for each variant allele is in black, the complementary regions of the primer sequence is in green, the variant alleles are in red and the INS are in deep red, grey and pink.

A novel strategy was devised for the multiplexed detection of SNPs and clustered mutations (Figs 2 and 3). In this approach each variant was amplified using an allele-specific PlexPrimer that was matched with the variant base at its 3T terminus and contained a mismatched base near the terminus to increase specificity. Each allele-specific PlexPrimer contained a different, unique INS which was in one of three conformations with respect to the original target templates., In the examples that follow the number of bases in the INS is ten and whilst all INSs are mismatched with respect to the target these ten bases may be either equal to (Planar Primers), greater than (Loop) or less than (Target Loop) the number of unbound bases in the target sequence (Fig 3i, 3ii and 3iii). As a consequence of using PlexPrimers with INS in different conformations, the 5’ regions (5T) of each PlexPrimer bound to different regions in the target. The resultant amplicons therefore had substantial sequence differences, which included the variant base, the 3T mismatched base, the INS, and the 5T binding region.

This strategy greatly reduced competition between primers, and cross binding between different allelic primer/amplicon pairs, even in highly multiplexed assays which targeted a group of tightly clustered allelic variants. Further, it enabled PlexPrime amplicons to be simultaneously detected in qPCR with allele-specific PlexZymes, each of which bound to the variant base and the complement of the INS, as well as downstream amplified target sequence (Fig 3iv, 3v and 3vi). In some scenarios the PlexZymes were designed to cleave different universal probes (Fig 2i), each labelled with a different fluorophore. In this format the variant alleles could be individually identified in multiplexed reactions in a single well. Alternatively PlexZymes were designed to cleave the same universal probe (Fig 2(ii)), which allowed many variants to be multiplexed and detected through a single fluorescence channel. In this fashion, allele-specific PlexPrimers were coupled with allele-specific PlexZyme detection in multiplex qPCR.

### Amplification and detection of SNPs in ADME genes

Multiplexed PlexPrime/PlexZyme assays were designed for 14 different SNPs within the ADME genes; of which twelve were bi-allelic, one was tri-allelic and one involved a deletion. Overall, characterisation of these SNPs involved discrimination of the full spectrum of alternate bases (G/A, G/T, G/C, A/T and C/T). The 14 ADME SNPs were analysed in 16 samples of gDNA using PlexPrime/PlexZyme assays and results were compared to commercial hydrolysis probe kits. The different gDNA samples showed homozygous and heterozygous genotypes. PlexPrime/PlexZyme assays showed 100% concordance (62/62) with results generated using hydrolysis probe assays (Table B in S1 File).

**Fig 4:**
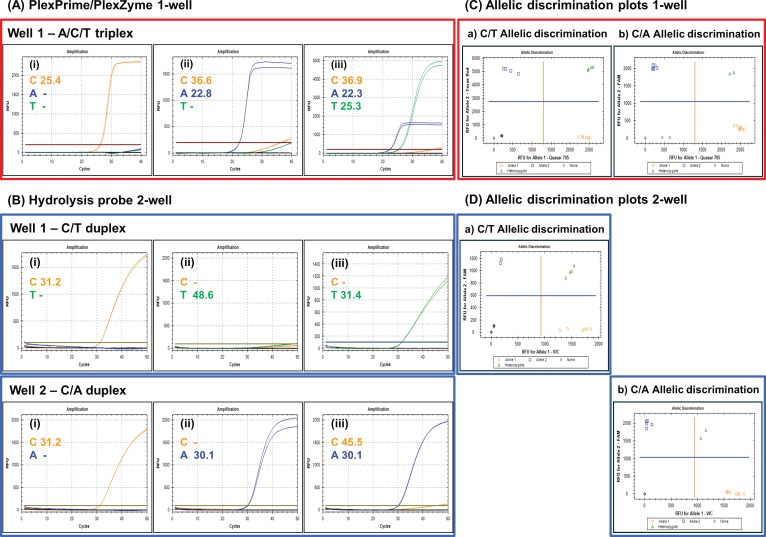
Comparison of PlexPrime/PlexZyme and hydrolysis probe assays for genotyping ADME SNPs showing real-time amplification plots and allelic discrimination graphs. (A) The PlexPrime/PlexZyme 1-well triplex assay reads C, A and T results in the Quasar 705, FAM and Texas Red channels, respectively. (B) The hydrolysis probe 2-well assay reads C and A in the VIC and FAM channels in well 1, and reads C and T in the VIC and FAM channels in well 2. The three samples used in these exemplary graphs are (i) 18855 which is homozygous for the C allele, (ii) 18608 which is homozygous for the A allele and (iii) 18970 which is heterozygous for alleles A and T. The Cq values for each allelic assay are shown on the amplification plot and the plotted graph for the C assay is orange, the A assay is blue and the T assay is green. The allelic discrimination plots display all of the samples listed in Table 1 for the SNP ABCB1 rs2032582; where (C) are the PlexPrime/PlexZyme plots derived from a single well and (D) are the Hydrolysis probe plots derived from two wells. The allelic discrimination plots have either a) Allele 1 in orange (C/C or C/A), Allele 2 in blue (T/T or A/T), Heterozygous in green (C/T) or None in black (NTC or A/A); or b) Allele 1 in orange (C/C or C/T), Allele 2 in blue (A/A or A/T), Heterozygous in green (C/A) or None in black (NTC or T/T).

Whilst duplex detection of two variant alleles at one SNP can be easily achieved with various chemistries, the discrimination of three or more alleles simultaneously in one multiplex reaction is far more challenging. A single well, multiplex PlexPrime/PlexZyme assay for concurrent detection of each of the three different alleles was designed to genotype the tri-allelic ADME SNP, rs2032582 in the ABCB1 gene (Fig 3, Table 2). The assay combined three allele-specific PlexPrimers which had Planar, Loop and Target Loop conformations (Figs 2 and 3). These were simultaneously interrogated in real-time using three allele-specific PlexZymes, each capable of cleaving a unique universal probe. Since the probes where labelled with different fluorophores, the three alleles were able to be individually detected in three separate channels (Fig 2(i)). The triplex PlexPrime/PlexZyme assay showed 100% concordance with the two duplex hydrolysis probe assays in 9/9 samples, with the advantage that the tri-allelic SNPs could be identified in one well as opposed to two wells required for the two duplex hydrolysis probe assays. An example of amplification plots is shown in Fig 4 for 3 samples; NA18855 which is homozygous for the C allele, NA18608 which is homozygous for the T allele and NA18970 which is heterozygous for the T and A alleles. The triplex PlexPrime/PlexZyme assay generated steep amplification curves for all alleles, consistent with efficient amplification, and also allowed unambiguous discrimination between all three alleles. The amplification curves generated by the two hydrolysis probe assays were less steep suggesting lower amplification efficiency; however, they still discriminate between alleles (Fig 4).

### Multiplexing clustered mutations

The superior capacity of PlexPrimers/PlexZymes for multiplexed mutation detection is further demonstrated in the MG 23S assay. This assay simultaneously detects *M*. *genitalium*, five mutations associated with azithromycin resistance, and an internal control. Specifically, this single well, 7-plex assay can simultaneously detect (i) the MgPa gene of *M*. *genitalium* in the first channel, (ii) any of five mutations present at positions 2058 and 2059 (*E*. *coli* numbering) of the 23S rRNA gene (A2058G, A2058C, A2058T, A2059G, and A2059C) in a second channel, and (iii) an internal control in a third channel. The internal control is non-homologous sequence which is added prior to extraction and is then co-extracted and co-amplified together with the targets. Any change in in either extraction and/or amplification efficiency will be reflected both in the control and in the target gene and mutations.

**Fig 5:**
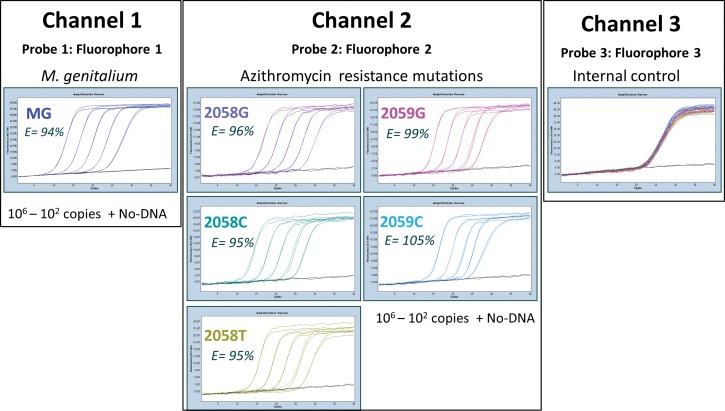
Amplification plots for the MG 23S assay. Analysis of serial dilutions (10^6^ to 10^2^ copies in a background of human gDNA) showing detection of *M*. *genitalium* (MgPa gene) in channel 1 and five 23S rRNA mutations associated with azithromycin resistance (2058G, 2058C, 2058T, 2059G and 2059C) in channel 2. An internal control for monitoring reaction efficiency is read in channel 3. No-DNA control reactions were run in parallel. The amplification efficiency (E) of each target is indicated.

In this multiplex assay, for the detection of the 23S rRNA mutations, five PlexPrimers targeting the five mutations were designed in the various formats; two had Loop and three had Target Loop conformations with 1, 6, 12, 17 and 23 bases unbound in the target sequence, respectively. The PlexPrimers were combined with allele-specific PlexZymes, all of which were designed to cleave the same common universal probe (illustrated in Fig 2ii). Analysis of serial dilutions demonstrated that all targets were detected robustly in multiplex with high efficiency; namely 94% for MgPa and between 95–105% for the 23S rRNA mutations (Fig 5 and Table C in S1 File). When a negative control reaction containing 10^6^ copies of the 23S rRNA wild type template was run it produced a non-specific background signal (Cq 21.1) which corresponds to a ΔCq of between 7.4 to 9.7 compared to Cq values for the same number of copies of each mutant template.

### Highly sensitive detection of deletion mutations

**Fig 6:**
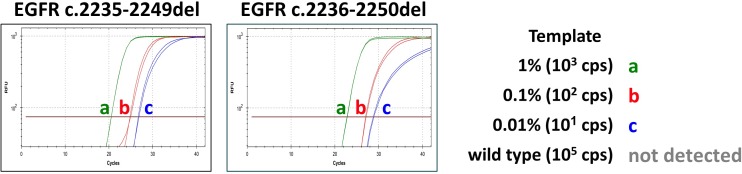
Amplification plots showing highly sensitive and specific detection of EGFR deletions. EGFR deletions c.2235-2249del and c.2236-2250del were diluted to a) 1% (10^3^ copies); b) 0.1% (10^2^ copies); and c) 0.01% (10 copies) in a background of 10^5^ copies of wild type alleles. A control reaction containing only 10^5^ copies of wild type alleles did not result in any signal.

The PlexPrime/PlexZyme strategy can also be used for highly sensitive detection of deletion mutations. A single PlexPrimer was designed to amplify the EGFR exon 19 deletion mutations, c.2235-2249del and c.2236-2250del. The performance of this assay was tested on a dilution series for both of these deletions, with results showing sensitive detection of 10 copies in a background of wild type DNA equivalent to 0.1% sensitivity with efficiencies (and linearities) of 92% (R^2^ = 0.996) and 97% (R^2^ = 0.998) for the c.2235-2249del and c.2236-2250del templates, respectively (Table 4). Further experimentation showed that as little as 10 copies of both deletions could be detected in a background of 10^5^ copies of wild type alleles. The assay detected the deletions present in only 0.01% of total DNA (Fig 6), with very high specificity as evidenced by the lack of any background signal from 10^5^ copies of wild type alone.

**Table 4:** Specific, sensitive detection of EGFR deletions using a single PlexPrimer which selectively amplified multiple deletions.

	EGFR c.2235-2249del mutant plasmid	EGFR c.2236-2250del mutant plasmid	EGFR Wild Type gDNA
**Cq 10**^**4**^ **copies (100%)**	17.3	18.5	nd
**Cq 10**^**3**^ **copies (10%)**	21.0	22.0	n/a
**Cq 10**^**2**^ **copies (1%)**	24.5	25.4	n/a
**Cq 10 copies (0.1%)**	27.9	28.7	n/a
**Efficiency (%)**	91.9	96.7	n/a
**R**^**2**^	0.99	0.99	n/a

nd—not detected. n/a–not applicable.

## Discussion

A new approach for the sensitive and specific, multiplexed amplification and detection of genetic variants has been demonstrated using PlexPrime amplification coupled with PlexZyme detection in qPCR. While qPCR is a rapid, inexpensive and highly sensitive molecular diagnostic tool, current technologies based on allele-specific primers or allele-specific probes alone are limited in their ability to detect variant alleles in multiplex. The unique design of the PlexPrime/PlexZyme system allows allele-specific priming to be coupled with allele-specific detection thus providing sensitive multiplex analysis of variants even when these are tightly clustered.

### SNP amplification and detection

The general capability of PlexPrimers to accurately amplify SNPs was demonstrated using genotyping assays for the ADME genes. These genes are important as they are involved in the absorption, distribution, metabolism and excretion of drugs, and polymorphisms can affect drug efficacy and toxicity. PlexPrime/PlexZyme results for 14 ADME SNPs were 100% concordant to commercial hydrolysis probe kits. Further, analysis of the tri-allelic SNP rs2032582 was achieved in a single triplex PlexPrime assay, whereas two duplex hydrolysis probe assays were required to genotype all three alleles. As such PlexPrimers can increase throughput and reduce costs since reagents are halved. Competition between allele-specific hydrolysis probes limits the ability to multiplex SNPs beyond duplex. In contrast, the design of the PlexPrimers significantly reduces competition at both the amplification step and the detection step, thus facilitating higher order multiplexed qPCR detection of SNPs or mutations.

The triplex ADME assay provides a demonstration of how this is achieved with allele-specific PlexPrimers, each of which has a unique INS that is configured to adopt a different conformation upon initial binding to the template. The unique INS either creates a loop within the primer (Loop), within the target (Target Loop) or is aligned parallel with respect to the target (Planar) (Fig 2i and Fig 3). Although the length ofthe different INSs is the same, the adoption of different conformations results in the 5T regions of each type of PlexPrimer binding to different upstream target sequences. The three PlexPrime amplicons differ along the entire length of the 5’ primer binding region; namely the 5T target sequences, the INS sequences, and the allele-specific 3T termini (Fig 2). This increases primer specificity and reduces primer competition. Further, the short 3T region (approximately 10 bases) puts considerable pressure for highly selective binding and extension from the terminus at the annealing temperature of the PCR. Finally, since allele-specific partzymes (PzA) target unique INSs within amplicons, competition between PlexZymes is eliminated and targets can be multiplexed without loss of efficiency. The PlexPrime/PlexZyme system of combined allele-specific priming and detection enables robust, specific multiplexing with advantages that have been clearly demonstrated compared to protocols using either allele-specific priming or allele-specific detection.

### Mutation detection in oncology

One field in which highly specific and highly sensitive mutation detection is of increasing clinical importance is oncology. Genes that activate specific signalling pathways can provide actionable targets for therapies, and companion diagnostic tests can determine if patients are likely, or unlikely, to benefit from treatment. For example, patients with metastatic colorectal cancer who have KRAS mutations would not be expected to respond to anti-EGFR therapies such as cetuximab, whereas patients with metastatic melanoma who have a BRAF mutation may be suitable for anti-BRAF therapies such as vemurafenib and dabrafenib.

High level, highly specific multiplexing of oncogene mutations using PlexPrime/PlexZyme technology has been reported on the Biocartis Idylla™ system [16]. The Idylla™ is a fully integrated and automated molecular diagnostic platform which can perform sample preparation, qPCR and reporting within two hours. In that study a single Idylla™ cartridge facilitated detection of 18 mutations; 13 mutations in the KRAS gene and 5 mutations in the BRAF gene. Results demonstrated excellent specificity and minimal cross-reactivity for all targets. The assay was evaluated on a set of colon cancer and melanoma FFPE samples and results were >96% concordant with those from sequencing.

Closer examination of the performance of that Idylla assay indicates that the PlexPrime/PlexZyme combination is not only easier to multiplex but is also inherently more specific than alternate techniques. Cross-reactivity was measured by calculating the difference in the number of cycles between specific amplification by a primer with its matched mutant target and the non-specific signal from other unmatched mutant templates. The PlexPrime assay, detecting 18 mutations in a single Idylla™ cartridge, showed excellent specificity and cross-reactivity for all KRAS targets, with delta Cq values of > 7 between mutants and > 12 between each mutant and the wild type [16]. An alternative ARMS-Scorpion assay, which requires seven parallel singleplex assays to analyse seven mutations in the G12 and G13 codon hotspots of KRAS [17] reported significant cross-reactivity between KRAS mutants which was observed within < 3 cycles in 1 case and within < 7 cycles in 3 cases). Since the basic difference between an ARMS primer and a PlexPrimer is the presence of the INS sequence, this observation lends support to the hypothesis that a short terminal sequence, which is a consequence of introducing the INS sequence, results in greater selective pressure for primers to only extend templates when they are terminally matched. This advantage has also been reported in DPO and SuperSelective primers which incorporate polydeoxyinosine linkers or non-complementary bridges respectively near the 3’ termini of the primers [6,7]. However, the PlexPrime/PlexZyme strategy has an additional unique advantage in that it has been demonstrated to support higher level multiplexing together with detection mediated by binding to the unique INSs.

PlexPrime is not only useful for detection of SNPs and somatic point mutations but can also detect other mutations such as deletions and insertions. EGFR deletion mutations are often found in patients with non-small cell lung cancer (NSCLC) and 48% of these are exon 19 deletion mutations [18]. There are many therapies targeted at EGFR, and exon 19 deletions confer greater sensitivity to EGFR tyrosine kinase inhibitors. In our study a single PlexPrimer/PlexZyme combination was designed to target both of the two most common deletions, c.2235-2249del and c.2236-2250del. This assay was shown to be exquisitely sensitive, detecting 10 copies of the deletion mutants in 10^5^ copies of wild-type, equivalent to 0.01% sensitivity.

An Italian group recently reported results wherein a cohort of lung cancer cytological specimens where analysed using the Idylla™ EGFR Mutation assay (Biocartis), which detects 53 different mutations (point mutations, insertions and deletions) using integrated PlexPrime/PlexZyme technology. The system yielded valid results in 74/76 (97.3%) samples, with a sensitivity of 100% percent (n = 32). Further, the system revealed two additional point mutations and two addition deletions in EGFR which had been not been detected by their standard reference methods (Fragment length electrogram and TaqMan). Their study concluded the Idylla assay and platform enabled very rapid decision making with high sensitivity, large reference range and ease of use even in less experienced laboratories [19]. Likewise, Solassol et al reported similar clinical utility and convenience reporting that the Idylla KRAS Mutation Test afforded simple, highly reliable and rapid routine determination of KRAS mutational status to guide colorectal therapy [20].

Greater sensitivity of mutation detection is becoming increasingly important for the analysis of cancer samples [21,22], and cellular heterogeneity of solid tumours has implications for the development of drug resistance. Subpopulations of cells with advantageous mutations, which are initially present in low abundance, may be selected for during treatment. This can lead to clonal expansion resulting in reduced progression-free survival times and recurrence of disease [23,24]. Therefore, more sensitive techniques which detect low prevalence mutations in heterogeneous tumour tissue samples may better predict patient response to a targeted therapy. Liquid biopsies have been shown to contain circulating tumour cells (CTCs) or cell-free tumour DNA (cfDNA) shed from the primary or metastatic tumours into blood. These samples offer a non-invasive method for earlier detection and serial sampling for disease monitoring [23], and are more likely to capture tumour heterogeneity. However, liquid biopsies also contain an abundance of non-tumour cells or DNA and thus highly sensitive techniques are required to detect the relatively rare CTCs and cfDNA. The very high sensitivity of PlexPrime demonstrated in our study makes it particularly well suited for detecting minor emerging clonal populations and/or for monitoring disease using liquid biopsies. The true power of combining PlexPrime and PlexZyme is evidenced in Biocartis’ Idylla oncology menu which includes three cartridges that collectively analyse 98 mutations; namely 21 mutations in a KRAS Test (CE-IVD); 25 Mutations in a NRAS-BRAF-EGFR Test (CE-IVD) and 52 mutations in a EGFR Mutation Assay (RUO); and further with the recent release of the liquid biopsy Idylla™ ctKRAS Mutation Assay (RUO).

### Rapid amplification and detection of clustered variants

In another application, PlexPrime/PlexZyme was used to stack the detection of five clustered mutations in a single channel on a standard qPCR machine. *M*. *genitalium* is an emerging sexually transmitted bacterium, and mutations in the 23S rRNA gene at positions 2058 and 2059 (*E*. *coli* numbering) have been associated with treatment failure and resistance to azithromycin, a macrolide antibiotic [25–27]. A single well 7-plex/3-channel assay was used to simultaneously detect *M*. *genitalium* (channel 1), 5 mutations associated with resistance to azithromycin (channel 2), plus an internal control (channel 3). Comparison of the single target mutation assays to performance in the 7-plex reaction over a 5-log range, showed a minor change in efficiency of 94% to 95%, 94% to 95%, 96% to 95%, 95% to 99% and 98% to 105% for A2058G, A2058C, A2058T, A2059G, A2059C, respectively. Further, detection of 40 copies for each target was similar and the cross-reactivity between mutant signal and wild type signal was always greater than 7 cycles.

The five 23S rRNA mutations are all associated with resistance to azithromycin; hence stacking the readout of the resistance mutations in a single well allows rapid screening in a scenario where knowledge of which specific individual mutation is not required. Software provided with the kit uses an algorithm based on the ΔCq between total *M*. *genitalium* (channel 1) and 23S rRNA signals (Channel 2) to automate sample result interpretation and ensure mutants are correctly called so as to avoid false positive results. This software has been validated on clinical specimens with results published in Tabrizi et al [26].

These types of multiplex assays have great potential to increase throughput in pathology laboratories which run a battery of qPCR-based assays. This assay has since been validated on several hundred urine and urogenital samples with very high clinical sensitivity and specificity [28].

## Conclusion

Although PlexPrimers could be used with other detection technologies such as hybridisation probes and Molecular Beacons, it is only when coupled with PlexZyme qPCR, that it uniquely enables the combination of allele-specific priming and allele-specific detection. This strategy enables superior multiplexing capacity with sensitive and specific detection, as demonstrated for SNPs and mutations. PlexPrime/PlexZyme qPCR is a rapid, inexpensive, robust, sensitive and specific method, making it a viable molecular diagnostic tool.

## Supporting Information

S1 File Table A. PCR primers, partzymes and probes for ADME SNP assays. Table B. PlexPrime/PlexZyme assays results for ADME SNPs. Table C. PlexPrime/PlexZyme assay results for amplification of MgPa and 23S rRNA genes in serially diluted samples.
(DOCX)
