# Supplementary material for: Superior Multiplexing Capacity of PlexPrimers Enables Sensitive and Specific Detection of SNPs and Clustered Mutations in qPCR
Source: PLoS One. 2017 Jan 23;12(1):e0170087. doi: 10.1371/journal.pone.0170087 (PMC5256879; doi:10.1371/journal.pone.0170087)
Supplement: S1 File — Table A. PCR primers, partzymes and probes for ADME SNP assays. Table B. PlexPrime/PlexZyme assays results for ADME SNPs. Table C. PlexPrime/PlexZyme assay results for amplification of MgPa and 23S rRNA genes in serially diluted samples. (DOCX) [file pone.0170087.s001.docx]

# S1 File

# Supporting Information for:

**Superior multiplexing capacity of PlexPrimers enables sensitive and specific detection of SNPs and clustered mutations in qPCR**

**Tan LY, Walker SM, Lonergan T, Lima N, Todd AV, Mokany E**

**Table A. PCR primers, partzymes and probes for ADME SNP assays**

| **dbSNP ID** | **Allele 1 & 2 components** | **Type of oligonucleotide** | **Sequence (5'to 3')** | **Modification*** |
| --- | --- | --- | --- | --- |
| ***Bi-allelic SNP assays (rs1058930, rs1065852, rs4149117, rs1801265, rs4986893, rs11572103, rs56161402, rs5030655, rs1058930, rs4244285, rs1801272)*** | | | | |
| Bi-allelic SNPs | 1 | Probe 1 | ACCGCACCTCguCCCCAGCTC | 5' FAM, 3' BHQ1 |
|  | 2 | Probe 2 | AAGGTTTCCTCguCCCCAGCTC | 5' Q705, 3' BHQ2 |
| rs1058930 | 1, 2 | Reverse primer | AAATCAAAATACTGATCTGTTGCTA |  |
|  |  | Partzyme B | GAGCTGGGGAGGCTAGCTATTGCTTCCTGATCAAAATGGA | 3' phosphate |
|  | 1 | Forward primer | ATCACTGGATGTTAACAATCCTCGATTCGAGTTGGACTTTCTC |  |
|  |  | Partzyme A | ATTCGAGTTGGACTTTCTCGACAACGAGAGGTGCGGT | 3' phosphate |
|  | 2 | Forward primer | CACCAAGCATCACTGGATGTTACGTTGGCTACGGACTTTCTG |  |
|  |  | Partzyme A | TTGGCTACGGACTTTCTGGACAACGAGAGGAAACCTT | 3' phosphate |
| rs1065852 | 1, 2 | Reverse primer | CTTCATGCCATGTATAAATGC |  |
|  |  | Partzyme B | GAGCTGGGGAGGCTAGCTCCAGGCCCCCTGCCACTG | 3' phosphate |
|  | 1 | Forward primer | CCTGATGCACCGGCGCCAAGATTCGAGAACACGCTTCC |  |
|  |  | Partzyme A | ATTCGAGAACACGCTTCCCAACAACGAGAGGTGCGGT | 3' phosphate |
|  | 2 | Forward primer | CGGCGCCAACGCTGGGCCATTGACAGACACGCTTCT |  |
|  |  | Partzyme A | ATTGACAGACACGCTTCTCAACAACGAGAGGAAACCTT | 3' phosphate |
| rs4149117 | 1, 2 | Reverse primer | GTAGCTGATAATAAATGGCTCAGA |  |
|  |  | Partzyme B | GAGCTGGGGAGGCTAGCTTTTACCACATTTCTTCATGGGATA | 3' phosphate |
|  | 1 | Forward primer | GTTGTCTCCTTATGGGAACTGGCGTTGGCTACGTATTTTGAGAT |  |
|  |  | Partzyme A | TTGGCTACGTATTTTGAGATCACAACGAGAGGTGCGGT | 3' phosphate |
|  | 2 | Forward primer | ATTGGAATTGGTTGTCTCCTTATGCACAATGATGGTATTTTGAGAG |  |
|  |  | Partzyme A | CACAATGATGGTATTTTGAGAGACAACGAGAGGAAACCTT | 3' phosphate |
| rs1801265 | 1, 2 | Reverse primer | ACTTACAAAGCAGTTCTTATCAGG |  |
|  |  | Partzyme B | GAGCTGGGGAGGCTAGCTTTCCACTTCGGCCAAGAAATTA | 3' phosphate |
|  | 1 | Forward primer | CCTGGCTTTAAATCCTCGAACAAGACATACTACAACTCAGC |  |
|  |  | Partzyme A | AGACATACTACAACTCAGCGACAACGAGAGGTGCGGT | 3' phosphate |
|  | 2 | Forward primer | CTTTAAATCCTCGAACACAAACTCCGTTGGCTACGCAACTCAGT |  |
|  |  | Partzyme A | TTGGCTACGCAACTCAGTGACAACGAGAGGAAACCTT | 3' phosphate |
| rs4986893 | 1, 2 | Reverse primer | CAATTTCTTAACTTGATGGAAAAA |  |
|  |  | Partzyme B | GAGCTGGGGAGGCTAGCTGGGGTGCTTACAATCCTGATG | 3' phosphate |
|  | 1 | Forward primer | AGTGGTTTCTCAGGAAGCAAAAAAGACATACTAACCTGGCTC |  |
|  |  | Partzyme A | ACATACTAACCTGGCTCCAGACAACGAGAGGTGCGGT | 3' phosphate |
|  | 2 | Forward primer | CTCAGGAAGCAAAAAACTTGGCGATTCGAGAATACCTGGCTT |  |
|  |  | Partzyme A | TCGAGAATACCTGGCTTCAGACAACGAGAGGAAACCTT | 3' phosphate |
| rs11572103 | 1, 2 | Reverse primer | TTGCTCTTACACGAAGTTACATTA |  |
|  |  | Partzyme B | GAGCTGGGGAGGCTAGCTGGAAGCAATCGATAAAGTCCCG | 3' phosphate |
|  | 1 | Forward primer | CTGATCTGTTGCTAATATCTTACCCGTTGGCTACCTCCATTTTAAT |  |
|  |  | Partzyme A | TTGGCTACCTCCATTTTAATCAACAACGAGAGGTGCGGT | 3' phosphate |
|  | 2 | Forward primer | ATCAAAATACTGATCTGTTGCTAATCACAATGATCCTCCATTTTCAA |  |
|  |  | Partzyme A | CAATGATCCTCCATTTTCAACAACAACGAGAGGAAACCTT | 3' phosphate |
| rs56161402 | 1, 2 | Reverse primer | AAAAGAATCCCTGATGTCATTC |  |
|  |  | Partzyme B | GAGCTGGGGAGGCTAGCTTATATTGCATATTTTACCTGAAACAA | 3' phosphate |
|  | 1 | Forward primer | CTTTTATGTCGTTCTTCAAAAGCAGACATACTATCAAGACTAC |  |
|  |  | Partzyme A | AGACATACTATCAAGACTACGACAACGAGAGGTGCGGT | 3' phosphate |
|  | 2 | Forward primer | TCGTTCTTCAAAAGCATCAACCTCGTTGGCTAATCAAGACCAT |  |
|  |  | Partzyme A | GTTGGCTAATCAAGACCATGACAACGAGAGGAAACCTT | 3' phosphate |
| rs5030655 | 1, 2 | Reverse primer | CCATCACCCACCGGAGTGGTT |  |
|  |  | Partzyme B | GAGCTGGGGAGGCTAGCTGTGACCGAGGAGGCCGC | 3' phosphate |
|  | 1 | Forward primer | TTGGGCCTGGGCAAGAAGTCTCAATACCATTGGAGCTGT |  |
|  |  | Partzyme A | CAATACCATTGGAGCTGTGGACAACGAGAGGTGCGGT | 3' phosphate |
|  | 2 | Forward primer | TTGGGCCTGGGCAAGAAGTCAGACATACTATGGAGCCGG |  |
|  |  | Partzyme A | GACATACTATGGAGCCGGGACAACGAGAGGAAACCTT | 3' phosphate |
| rs1058930 | 1, 2 | Reverse primer | AAATCAAAATACTGATCTGTTGCTA |  |
|  |  | Partzyme B | GAGCTGGGGAGGCTAGCTATTGCTTCCTGATCAAAATGGA | 3' phosphate |
|  | 1 | Forward primer | ATCACTGGATGTTAACAATCCTCGATTCGAGTTGGACTTTCTC |  |
|  |  | Partzyme A | ATTCGAGTTGGACTTTCTCGACAACGAGAGGTGCGGT | 3' phosphate |
|  | 2 | Forward primer | CACCAAGCATCACTGGATGTTACGTTGGCTACGGACTTTCTG |  |
|  |  | Partzyme A | TTGGCTACGGACTTTCTGGACAACGAGAGGAAACCTT | 3' phosphate |
| rs4244285 | 1, 2 | Reverse primer | AATAAATTATTGTTTTCTCTTAGATA |  |
|  |  | Partzyme B | GAGCTGGGGAGGCTAGCTGAAATAATCAATGATAGTGGGAAAT | 3' phosphate |
|  | 1 | Forward primer | TTTCCATAAAAGCAAGGTTTTTAAGAGACATACTCTGGGTTGCC |  |
|  |  | Partzyme A | ACATACTCTGGGTTGCCGGACAACGAGAGGTGCGGT | 3' phosphate |
|  | 2 | Forward primer | TAAAAGCAAGGTTTTTAAGTAATTTGCACAATGATGATGGGTTGCT |  |
|  |  | Partzyme A | CAATGATGATGGGTTGCTGGACAACGAGAGGAAACCTT | 3' phosphate |
| rs1801272 | 1, 2 | Reverse primer | CGCCACCCTGCGGGACTTC |  |
|  |  | Partzyme B | GAGCTGGGGAGGCTAGCTGGCGTCGATGAGGAAGCC | 3' phosphate |
|  | 1 | Forward primer | GCACTCGGGGTCCCCTGCTAGACATACTAGCCCCTGA |  |
|  |  | Partzyme A | AGACATACTAGCCCCTGAGACAACGAGAGGTGCGGT | 3' phosphate |
|  | 2 | Forward primer | GGTCCCCTGCTCACCGCCACACAATGATGGCCCCTGT |  |
|  |  | Partzyme A | CACAATGATGGCCCCTGTGACAACGAGAGGAAACCTT | 3' phosphate |
| ***Bi-allelic SNP assays (rs1056836, rs316019, rs1799853)*** | | | | |
| Bi-allelic SNPs | 1 | Probe 3 | ACCGCACCTCguCCCCAGCTC | 5' FAM, 3' BHQ1 |
|  | 2 | Probe 4 | AAGGTTTCCTCguCCCTGGGCA | 5' HEX, 3' BHQ1 |
| rs1056836 | 1, 2 | Reverse primer | GGTCAGGTCCTTGTTGATGAGG |  |
|  |  | Partzyme B | TGCCCAGGGAGGCTAGCTGTGGCCTAACCCGGAGAACT | 3' phosphate |
|  | 1 | Forward primer | TTTGTCAACCAGTGGTCTGTGACGTTGGCTACCATGACCGAG |  |
|  |  | Partzyme A | GCTACCATGACCGAGTGAAACAACGAGAGGTGCGGT | 3' phosphate |
|  | 2 | Forward primer | CTGTGGTTTTTGTCAACCAGTGAGACATACTACATGACCGAC |  |
|  |  | Partzyme A | CATACTACATGACCGACTGAAACAACGAGAGGAAACCTT | 3' phosphate |
| rs316019 | 1, 2 | Reverse primer | TTTTATTCCAAATGGACTTACCAG |  |
|  |  | Partzyme B | TGCCCAGGGAGGCTAGCTTGCCCAACTTCTTCTTCTTGCT | 3' phosphate |
|  | 1 | Forward primer | CTTCCTCACTGGAGGTGGTTGAGACATACTTGTTCACAGATT |  |
|  |  | Partzyme A | ACATACTTGTTCACAGATTCTCACAACGAGAGGTGCGGT | 3' phosphate |
|  | 2 | Forward primer | TTACGCACTTCCTCACTGGAGCACAATGATCTTCACAGCTG |  |
|  |  | Partzyme A | AATGATCTTCACAGCTGCTCACAACGAGAGGAAACCTT | 3' phosphate |
| rs1799853 | 1, 2 | Reverse primer | GGTCAGTGATATGGAGTAGGG |  |
|  |  | Partzyme B | TGCCCAGGGAGGCTAGCTTTCAAGAGGAAGCCCGCTGC | 3' phosphate |
|  | 1 | Forward primer | TTGGGATGGGGAAGAGGAGCCACAATGATGTTGAGGTCC |  |
|  |  | Partzyme A | CAATGATGTTGAGGTCCGTGACAACGAGAGGTGCGGT | 3' phosphate |
|  | 2 | Forward primer | CTGCGGAATTTTGGGATGGGGTTACCTGAAATTGAGGTCT |  |
|  |  | Partzyme A | TACCTGAAATTGAGGTCTGTGACAACGAGAGGAAACCTT | 3' phosphate |

* Fluorophores used to label Probes are 6-FAM (FAM), Texas Red (TxR), Quasar 705 (Q705) and HEX. Quenchers attached to probes are Black Hole Quencher 1 and 2 (BHQ1 and BHQ2), Iowa Black^®^ FQ (FQ) and Iowa Black^®^ RQ (RQ).

Ribonucleotide bases are in lower case

**Table B. PlexPrime/PlexZyme assays results for ADME SNPs.**

| Gene | dbSNP | RefSNP Alleles* | Sample | Result^ |
| --- | --- | --- | --- | --- |
| TPMT | rs56161402 | C/T (S) | NA11830 | C/C |
|  |  |  |  |  |
| CYP2C9 | rs1799853 | C/T (S) | NA18992 | C/C |
|  |  |  |  |  |
|  |  |  | NA12248 | C/T |
|  |  |  |  |  |
|  |  |  | IM-9 | C/T |
|  |  |  |  |  |
| CYP2C19 | rs4244285 | A/C/G (S) | NA12891 | A/A |
|  |  |  |  |  |
|  |  |  | Calu-1 | A/A |
|  |  |  |  |  |
|  |  |  | NA11830 | G/G |
|  |  |  |  |  |
|  |  |  | NA18970 | G/G |
|  |  |  |  |  |
|  |  |  | NA18855 | A/G |
|  |  |  |  |  |
|  |  |  | NA19003 | A/G |
|  |  |  |  |  |
| CYP2C19 | rs4986893 | A/G (S) | NA18970 | G/G |
|  |  |  |  |  |
|  |  |  | NA18992 | G/G |
|  |  |  |  |  |
|  |  |  | NA18573 | A/G |
|  |  |  |  |  |
|  |  |  | NA18948 | A/G |
|  |  |  |  |  |
| CYP2D6 | rs5030655 | -/T (A) | NA11830 | T/T |
|  |  |  |  |  |
| CYP2D6 | rs1065852 | C/T (A) | NA12248 | C/C |
|  |  |  |  |  |
|  |  |  | NA18608 | C/C |
|  |  |  |  |  |
|  |  |  | NA18855 | C/C |
|  |  |  |  |  |
|  |  |  | NA18992 | C/C |
|  |  |  |  |  |
|  |  |  | NA19003 | C/C |
|  |  |  |  |  |
|  |  |  | NA18948 | T/T |
|  |  |  |  |  |
|  |  |  | IM-9 | T/T |
|  |  |  |  |  |
|  |  |  | NA06993 | C/T |
|  |  |  |  |  |
|  |  |  | NA11830 | C/T |
|  |  |  |  |  |
|  |  |  | NA18573 | C/T |
|  |  |  |  |  |
|  |  |  | NA18871 | C/T |
|  |  |  |  |  |
|  |  |  | NA18970 | C/T |
|  |  |  |  |  |
| SLC22A2 | rs316019 | G/T (A) | NA18608 | G/G |
|  |  |  |  |  |
|  |  |  | NA18855 | G/G |
|  |  |  |  |  |
|  |  |  | IM-9 | G/T |
|  |  |  |  |  |
| SLCO1B3 | rs4149117 | G/T (S) | NA18573 | G/G |
|  |  |  |  |  |
|  |  |  | NA18948 | G/G |
|  |  |  |  |  |
|  |  |  | NA18855 | T/T |
|  |  |  |  |  |
|  |  |  | NA18992 | T/T |
|  |  |  |  |  |
|  |  |  | NA06993 | G/T |
|  |  |  |  |  |
|  |  |  | NA18608 | G/T |
|  |  |  |  |  |
| CYP2C8 | rs11572103 | A/T (A) | NA11830 | T/T |
|  |  |  |  |  |
| CYP2C8 | rs1058930 | A/C/G (S) | NA18871 | C/C |
|  |  |  |  |  |
|  |  |  | NA18948 | C/C |
|  |  |  |  |  |
|  |  |  | Calu-1 | G/G |
|  |  |  |  |  |
|  |  |  | NA12248 | C/G |
|  |  |  |  |  |
|  |  |  | NA12891 | C/G |
|  |  |  |  |  |
| CYP2A6 | rs1801272 | A/T (A) | NA11830 | A/A |
|  |  |  |  |  |
| DPYD | rs1801265 | C/T (A) | NA18855 | C/C |
|  |  |  |  |  |
|  |  |  | NA18871 | C/C |
|  |  |  |  |  |
|  |  |  | NA18608 | T/T |
|  |  |  |  |  |
|  |  |  |  |  |
|  |  |  | Calu-1 | T/T |
|  |  |  |  |  |
|  |  |  | HT-29 | C/T |
|  |  |  |  |  |
|  |  |  | SW480 | C/T |
|  |  |  |  |  |
| CYP1B1 | rs1056836 | C/G (A) | NA18871 | C/C |
|  |  |  |  |  |
|  |  |  | SW480 | C/C |
|  |  |  |  |  |
|  |  |  | NA12891 | G/G |
|  |  |  |  |  |
|  |  |  | NA18948 | G/G |
|  |  |  |  |  |
|  |  |  | NA12248 | C/G |
|  |  |  |  |  |
| ABCB1 | rs2032582 | A/G/T (A) | NA18855 | C/C |
|  |  |  |  |  |
|  |  |  | NA18871 | C/C |
|  |  |  |  |  |
|  |  |  | NA18573 | A/A |
|  |  |  |  |  |
|  |  |  | NA18608 | A/A |
|  |  |  |  |  |
|  |  |  | NA19003 | T/C |
|  |  |  |  |  |
|  |  |  | NA06993 | C/A |
|  |  |  |  |  |
|  |  |  | NA18970 | T/A |
|  |  |  |  |  |

*(S) = sense strand and (A) = antisense strand

^Alleles as determined by PlexPrime/PlexZyme assay and hydrolysis probe assay

**Table C. PlexPrime/PlexZyme assay results for amplification of MgPa and 23S rRNA genes in serially diluted samples.**

|  | Copies of template | | | | | | | | | |  | |
| --- | --- | --- | --- | --- | --- | --- | --- | --- | --- | --- | --- | --- |
|  | 10^6^ | | 10^5^ | | | 10^4^ | 10^3^ | | | 10^2^ | Efficiency (%) |  |
| MgPa template^ | 10.0 | | 13.6 | | | 17.0 | 20.6 | | | 23.9 | 94 |  |
| A2058T template | 12.8 | | 16.2 | | | 19.7 | 23.2 | | | 26.0 | 95 |  |
| A2058C template | 11.4 | | 14.9 | | | 18.4 | 21.7 | | | 24.5 | 95 |  |
| A2058G template | 13.7 | | 17.1 | | | 20.6 | 23.6 | | | 26.8 | 96 |  |
| A2059C template | 13.6 | | 16.9 | | | 20.0 | 22.6 | | | 24.7 | 105 |  |
| A2059G template | 12.4 | | 15.8 | | | 19.2 | 22.4 | | | 24.8 | 99 |  |
| Wild type template | 21.0 | | - | | | - | - | | | - |  |  |
| Internal Control^ | - | | - | | | - | - | | | 21.4 (+/- 0.3) |  |  |
| ^ Averaged Cq value | |  | |  |  | | |  |  |  |  |  |
